# Tuberculosis in the era of infection with the human immunodeficiency virus: assessment and comparison of community knowledge of both infections in rural Uganda

**DOI:** 10.1186/1472-698X-12-36

**Published:** 2012-12-20

**Authors:** Ashley Wynne, Gian S Jhangri, Solina Richter, Arif Alibhai, Tom Rubaale, Walter Kipp

**Affiliations:** 1 School of Public Health, University of Alberta, 3-300 Edmonton Clinic Health Academy, 11405 87th Ave, Edmonton, Alberta, T6G 1C9, Canada; 2 Faculty of Nursing, University of Alberta, 5-269 Edmonton Clinic Health Academy, 11405 87th Ave, Edmonton, Alberta, T6G 1C9, Canada; 3 Health Department, Kabarole District Administration, Fort Portal, Uganda

**Keywords:** Tuberculosis, Human immunodeficiency virus, Community knowledge, Uganda

## Abstract

**Background:**

In Uganda, despite a significant public health burden of tuberculosis (TB) in the context of high human immunodeficiency virus (HIV) prevalence, little is known about community knowledge of TB. The purpose of this study was to assess and compare knowledge about TB and HIV in the general population of western Uganda and to examine common knowledge gaps and misconceptions.

**Methods:**

We implemented a multi-stage survey design to randomly survey 360 participants from one district in western Uganda. Weighted summary knowledge scores for TB and HIV were calculated and multiple linear regression (with knowledge score as the dependant variable) was used to determine significant predictors. Six focus group discussions were conducted to supplement survey findings.

**Results:**

Mean (SD) HIV knowledge score was 58 (12) and TB knowledge score was 33 (15), both scores out of 100. The TB knowledge score was statistically significantly (p < 0.001) lower. Multivariate regression models included age, sex, marital status, education, residence, and having a friend with HIV/TB as independent variables. TB knowledge was predicted by rural residence (coefficient = −6.27, 95% CI: -11.7 to −0.8), and age ≥45 years (coefficient = 7.45, 95% CI: 0.3-14.6). HIV knowledge was only predicted by higher education (coefficient = 0.94, 95%CI: 0.3-1.6). Focus group participants mentioned various beliefs in the aetiology of TB including sharing cups, alcohol consumption, smoking, air pollution, and HIV. Some respondents believed that TB was not curable.

**Conclusion:**

TB knowledge is low and many misconceptions about TB exist: these should be targeted through health education programs. Both TB and HIV-infection knowledge gaps could be better addressed through an integrated health education program on both infections, whereby TB program managers include HIV information and vice versa.

## Background

Globally, TB is one of the most common infections based on the following numbers: In 2010, there were 8.8 million new tuberculosis (TB) cases and 1.4 million deaths caused by TB worldwide [1]. Much of this burden co-exists with human deficiency virus (HIV) in high HIV-prevalence countries in sub-Saharan Africa such as Uganda [2]. TB still constitutes a major challenge in Uganda; in 2010, TB incidence in Uganda was estimated at 209/100,000 population per year, or 70,000 new TB cases. Fifty four percent of new TB cases (with known HIV status) were in HIV positive individuals [1].

This data show that TB is still an overarching public health issue both globally and in Uganda, in spite of effective methods available to curb the spread of TB such as the treatment of smear positive TB cases. The cooperation and understanding of patients, family members and the community at large is required in order to effectively deal with TB, as has been shown for mental illness [3]. One example was reported in a paper published by Edington [4], where South African TB patients believed that while on TB treatment they must abstain from sex. This belief negatively affected adherence to medication. The authors concluded that there was a need for health care workers to learn about local beliefs in order to provide more relevant medical care and health education. Furthermore, perceptions around disease and ill health, not just of patients but of family, friends, and general community members, have been identified as important factors influencing health seeking behaviour, including treatment adherence in persons with TB [5]. In addition to adherence, perceived knowledge on the causes of TB has been found to influence transmission of the disease, and certain beliefs have resulted in failure to recognize symptoms and thus delayed diagnosis and treatment [6]. It has also been highlighted that health care workers are often not aware of the knowledge and beliefs about TB within the communities they serve [4]. When lack of congruence in beliefs and practices exists between individuals and healthcare providers in a community, poorer patient adherence and treatment outcomes have been shown to occur [3]. Taking this into account, it is imperative to assess and evaluate the local TB knowledge and perceptions of the community. This information will allow district health care workers to better tailor health messages and TB program services to the specific needs of the patients, likely resulting in better program outcomes.

Despite the importance of understanding community knowledge, relatively few studies have investigated the local knowledge of TB. A literature review was completed with the assistance of a health sciences librarian at the University of Alberta. Databases searched included Global Health, Medline, Psychinfo, Web of Science, and Academic Search Complete. Search headings and title searches used were knowledge level/ knowledge/ health behaviour/ perception/ awareness/ care seeking/ health seeking/ understandings/ conceptualisations. These results were geographically limited using the subject headings Africa south of the Sahara/ East Africa/ Africa/ Anglophone Africa/ Uganda. Finally, the search was narrowed to include only articles relating to tuberculosis and HIV or Acquired Immune Deficiency Syndrome (AIDS). Grey literature from the World Health Organisation and the Ugandan Ministry of Health was also included. Seven articles related directly to our topic, two conducted in South Africa, and one each in Tanzania, Rwanda, Kenya, Ethiopia, and Uganda [4,6-11]. All studies generally found a very low knowledge of TB in the community. A major variation in TB knowledge even within one country was reported from South Africa [4,7], indicating that the results from one study cannot be generalised even to other areas of the same country. In Uganda, only one study to date has investigated the local knowledge of TB. It was conducted in Eastern Uganda and found that respondents held multiple TB aetiologies including sharing utensils, heavy labour, smoking, bewitchment, and hereditary transmission. There was also a strong assumption of HIV co-infection in TB patients [11]. In order to generate information of communities’ knowledge of TB from western Uganda (whose cultural context is different from eastern Uganda), and to improve TB prevention and treatment outcomes, we conducted this study from September to December 2010 in Kabarole district. In our study, we also assessed community knowledge of HIV infection (which has received much more attention compared to TB and which is associated with TB) to be able to compare and contrast the knowledge in the community of these two high priority infections.

### Objectives of the study

The objectives of this study were:

1. To assess and compare the local community knowledge of TB and HIV infection in rural western Uganda;

2. To determine predictors of high knowledge of TB and HIV infection;

3. To identify specific misconceptions and beliefs that may influence the utilization and effectiveness of TB services.

## Methods

### Study design and recruitment of participants

This was a cross-sectional sequential mixed methods study with a quantitative and a qualitative study component. The study population was the total general adult population of Kabarole district in western Uganda, from which a random sample was drawn. Participants for the quantitative survey were selected using four levels of cluster random sampling (number selected): sub-county (6), parishes (18), villages (18), and households (360). One person was selected from each household. A total of 360 residents of Kabarole district agreed to participate. For the quantitative survey, a sample size of 360 was determined to be able to detect a 15% difference in the knowledge score of TB and HIV with a 0.05 significance level (two tailed test and a power of 80%). For the qualitative study component, the participants were not part of the quantitative survey, but were selected using convenience sampling from the same sub-counties (but different villages) as the survey sample. Six focus group discussions were conducted

### Description of study area

The study was conducted in Kabarole district (population estimate 455,000 in 2010, land area 4,800 km^2^). The district health services consist of 47 health units with 75% of the population being less than 5 kilometres away from a unit [12]. The district TB services are organized by the District TB Officer and are decentralized and offered through the various health units. Services are coordinated under the National TB Control Program which officially follows WHO recommendations on Directly Observed Treatment Short Course (DOTS), though in practice resource constraints prevent full DOTS program implementation in some health units. In reported new smear positive cases, the cure rate (those who had a smear negative result at the end of treatment) was 15%, treatment completion rate was 60% and the defaulter rate was 12% in the Western Zone of Uganda, which includes our study area, as reported by the National TB/Leprosy programme Uganda in 2009 (unpublished report).

### Study instruments

A questionnaire was developed to collect information on demographics and knowledge about HIV and TB transmission, symptoms, prevention, and treatment. As no standard questionnaire was available and suitable to our study objectives, we developed our own questionnaire in collaboration with local partners. This ensured a balanced scope of knowledge questions as well as ensured appropriateness and comprehensiveness of the questions in the local context. The questionnaire covered additional items related to stigma and TB/HIV co-infection. The total number of knowledge questions regarding TB and HIV were 17 questions. The questions were open-ended and formulated in a simple way to make them understandable to our participants. The most important questions for both infections were: “How can someone get TB?”; “What are the main symptoms of TB?”; “Is it possible for TB to be completely cured?”; “How can someone get HIV?”; ; “What are the things a person can do to avoid getting HIV?”; “Is it possible for HIV to be completely cured?”

Based on these questions, the maximum possible score was 14 for the TB knowledge and 17 for the HIV knowledge. Each respondent’s knowledge scores were expressed as a percentage of the total possible scores. The questionnaire was pre-tested in the field. The reliability of the questionnaire was assessed by re-administering the questionnaire after seven days to a random sample of 10 respondents. The comparison of responses was used to calculate the agreement of responses between the survey and the re-test. The agreement in responses for all questions of the questionnaire was 86%. For TB knowledge questions agreement was 91%, and for HIV-infection knowledge questions it was 95%. The HIV status and TB status (or history) of participants was not assessed, therefore this sample neither excludes nor includes HIV or current or former TB patients.

### Data collection and analysis

For the quantitative part, four local research assistants administered the questionnaire through an interview in the homes of the respondents. All were experienced interviewers and were thoroughly trained on the questionnaire. The interviews were conducted in the local language (Rutoroo) and lasted about 30 minutes. All completed interview sheets were checked for completeness by the first author. Summary knowledge scores were calculated by combining the correct responses from the knowledge questions and weighing it according to the importance of the question. All analyses were conducted using Stata 11 and controlled for the multistage survey design. Stata’s survey feature was used to take into account the number selected versus available sampling units at each level of sampling to account for different sample pools at each level [13]. Descriptive statistics were used to summarize each questionnaire item. Two-tailed student t-tests or Analysis of Variance (ANOVA) were used to compare TB and HIV knowledge scores by demographic characteristics. Multiple linear regression was used to model the summary scores for HIV knowledge and TB knowledge separately. Relevant variables of interest were purposefully selected and included in the model. The significance level was set at 0.05.

Six focus group discussions (FGD) were conducted to further explore issues arising from the survey. Three FGDs were held with women only and three with men only. Two FGDs (one with men, one with women) were held with urban participants from the district capital and four FGDs were held with rural dwellers. The FGDs were conducted by the first author and a research assistant in the local language (for demographic details see Table 1):

**Table 1:** Demographic description of focus group participants.

**Focus group**	**Gender**	**Location**	**Number of participants**	**Age**		
				**Mean**	**Median**	**Range**
1	Male	Fort Portal	11	30	25	(19–64)
2	Female	Fort Portal	11	23	24	(18–28)
3	Male	Bukuuku	9	33	23	(18–70)
4	Female	Bukuuku	11	33	30	(19–52)
5	Male	Kibiito	11	40	38	(22–78)
6	Female	Kibiito	8	36	36	(18–58)

We developed a topic list jointly with our local team members and key informants. Transcripts were analyzed using thematic analysis to identify, code, and categorize the data. Themes were developed from these categories. A sub-sample of transcripts was re-coded by the same researcher, and a sub-set was also coded by an independent researcher to ensure inter-rater reliability (agreement in the coding tree was established) and to contribute to the trustworthiness of the study. Qualitative results were presented and discussed with participants and key informants (member checking) to confirm and validate the themes. The focus group discussions were conducted in the local language Rutoroo.

The responses from the questionnaire in the local language were immediately translated into English by the research assistants in the field and checked at the end of the day by the first author. The qualitative transcripts from the focus group discussions in the local language were also translated into English. A sub set of these transcripts were translated by an independent translator and the two English versions were compared to assess the accuracy of the translation.

### Ethical approval

This study received ethical approval in Canada from the University of Alberta Human Research Ethics Board, Health Panel, and in Uganda from Makerere University School of Public Health’s Internal Review Board and the Uganda National Council for Science and Technology. Participants were given information and a detailed explanation of the study by a trained research assistant in the local language. Informed consent was provided by signature or thumbprint on the consent form.

## Results

Of the 360 survey respondents 50.7% (n = 182) were female, mean age was 33 years (median 29, range 18–84 years). Other demographic information for the survey respondents can be found in Table 2.

**Table 2:** Demographic characteristics of survey participants.

**Variable**	**n**	**(%)**
**Age in years**		
18-24	116	32.2
25-44	174	48.3
> = 45	70	19.4
**Sex**		
Male	177	49.3
Female	182	50.7
**Type of house**		
Permanent	46	12.9
Semi-permanent	312	87.2
**Education level**		
None or Primary	238	66.3
Some Secondary or higher	121	33.7
**Marital status**		
Single	140	38.9
Living with partner or ever married	220	61.1
**Residence**		
Urban	60	16.7
Rural	300	83.3

Three “women only” focus group discussions contained a total of 30 women, and three “men only” focus group discussions included a total of 31 men. Male participants in the focus group discussions were slightly older (mean 34 years, median 28 years, range 18–78 years) than female participants (mean 30 years, median 26 years, and range 18–58 years). Two focus group discussions were conducted in an urban area (one male, one female). Urban participants were younger (mean 26 years, median 25 years, range 18–64 years) than rural participants (mean 36 years, median 31 years, and range 18–78 years).

### TB knowledge

Overall TB knowledge was limited. Three hundred forty three participants (95.3%) mentioned that cough is one of the main signs and symptoms of TB, but of the other two classic TB symptoms weight loss and night sweats, only 91 people (25.3%) mentioned weight loss, and only 10 people mentioned anything that could be interpreted as “night sweats” (responses included fever, sweating, evening fevers, evening sweats, and “evening malaria”). When asked how someone can get TB (as an open ended question allowing multiple responses), 49 people (13.7%) mentioned that it was an airborne disease, and 62 (17.3%) people said that you can get it from being near a patient. The most common form of transmission mentioned was through saliva, usually by sharing cups (263 participants, 73.3%). Fifty eight people (16.2%) mentioned that someone could get TB from smoking and/or drinking, 35 people (9.8%) stated that TB was hereditary, 35 (9.8%) mentioned other ways of getting TB (for example from uncooked milk or meat, from working in factories, from hard labour), and 21 (5.9%) were unsure how TB is transmitted. Two hundred eighty nine people (80.5%) knew that TB can be cured.

The mean TB knowledge summary percentage score was 34 (SD 15, range 0–79). Univariate and multiple linear regression analysis of TB knowledge considered age, education, type of house, sex, marital status, place of residence, and knowing someone who has had TB as independent variables. For both univariate and multivariate regression, older age (≥45 years) was statistically significantly associated with higher TB knowledge, and urban residence was statistically significantly associated with lower TB knowledge (see Table 3).

**Table 3:** Univariate and multivariate linear regression analysis of TB knowledge score

		**Univariate analysis**		**Multivariate analysis**	
**Variable**		**slope (95% CI)**	**p**	**slope (95% CI)**	**p**
Age	18-24	1.0		1.0	
	25-44	1.33 (−4.47, 7.12)	0.582	1.88 (−3.43, 7.19)	0.404
	> = 45	6.76 (0.27, 13.26)	**0.044**	7.45 (0.30, 14.60)	**0.044**
Education	Primary or less	1.0		1.0	
	Secondary or higher	1.08 (−3.63, 5.80)	0.580	3.07 (−2.03, 8.18)	0.183
House**	Mud walled	1.0		-	
	Concrete walled	−1.75 (−7.69, 4.18)	0.482	-	
Sex	Male	1.0		1.0	
	Female	−0.43 (−6.34, 5.51)	0.859	−0.31 (−6.08,5.45)	0.894
Marital status	Single	1.0		1.0	
	Ever married	1.17 (−5.27,7.62)	0.660	−0.49 (−6.77, 5.79)	0.850
Residence	Rural	1.0		1.0	
	Urban	−6.02 (−11.37, -0.68)	**0.034**	−6.27 (−11.73,-0.81)	**0.032**
Has a friend who has had TB	No	1.0		1.0	
	Yes	1.04 (−4.59,6.67)	0.654	0.63 (−4.47, 5.74)	0.762

**Not included in model because of collinearity.

**Bold** indicates statistically significant values (p <0.05).

Focus group participants discussed general population knowledge about TB, different types of TB symptoms, and transmission. Respondents reported a general lack of knowledge about TB amongst their village members. A lack of awareness was linked to delays in seeking treatment due to not recognizing the symptoms of TB. When asked why people were dying of TB despite treatment being available, one respondent stated:

"“For me I think our people lack information. You find someone coughing without knowing that it’s TB.” (urban male)"

There was an expressed perception that TB isn’t curable. Participants mentioned either directly that TB doesn’t have a cure, or that their fellow villagers believed that TB has no cure. One respondent stated:

"“The government announced that there is no complete cure for TB, that one we know.” (rural male)"

Distinctions were also made between types of TB that are not curable, for example “inherited” TB cannot be cured, or TB that has progressed very far cannot be cured. It was also believed that TB cannot be cured in HIV patients. One explanation given was because HIV destroys all the white blood cells so even with the help of strong drugs the body cannot fight TB.

People believed that TB is transmitted through saliva by sharing utensils, cups, plates, and food stuffs. For example, one respondent noted that:

"“When a TB patient spits and another comes close to it [he/she] gets TB. Then sharing things like cups, forks you also get it.” (urban female)"

The idea of TB being transmitted through sharing cups is especially mentioned in the context of sharing cups when drinking alcohol. The situation of passing around a common cup at a local bar is associated with TB transmission. For example, one respondent stated:

"“TB spreads easily to those people who drink local booze because they share straws among themselves.” (rural male)."

In addition to alcohol, smoking was also understood as a way how TB is transmitted.

TB was also associated by respondents with being an environmental air pollutant. TB can be contracted from smoky fires in local houses that don’t have good chimneys and where the kitchen and bedrooms are together.

"“Most especially we have kitchens in the same house where people sleep, so the smoke is too much. [The] lungs develop something like feathers which causes that person to cough, cough and cough.” (urban male)"

"“People who work mostly in industries and factories are vulnerable.” (urban male)"

There was also an understanding of TB being inherited from one’s parent through blood, that it runs in a family, as one respondent explained:

"“Some diseases we inherit from the blood of our parents like TB, that TB can be inherited.” (rural male)"

TB was correctly associated by respondents with a very serious set of symptoms including very severe and prolonged cough, often associated with coughing up or vomiting blood. Less severe symptoms were assumed to be a “normal cough”, and so people didn’t seek medical attention. As one participant explained:

"“Yes, asthma and other things, regular coughs. I see that the reason leading to many people dying of TB [is that] they assume it’s a normal cough yet it’s TB.” (rural female)"

Abnormal weight loss was also understood as a symptom of TB, but not independent of cough. There was almost no mention of night sweats (the only person who mentioned it was a nurse) and no discussion of having weight loss/ night sweats without the cough.

### HIV knowledge

Three hundred and fifty people (97.2%) mentioned sexual transmission when asked how someone can get HIV. Other responses were sharing sharp instruments (146, 40.6%), blood transfer (68, 18.9%), mother to child transmission (49, 13.6%) and having many partners (34, 9.4%). In order to assess knowledge of HIV prevention, respondents were asked “What are the things a person can do to avoid getting HIV?” Three hundred and twelve people mentioned condoms (86.7%), 230 mentioned abstinence (63.9%), and 166 mentioned ‘be faithful’ (46.1%). Three hundred and forty people (94.7%) knew that HIV cannot be completely cured.

The mean HIV knowledge summary percentage score was 58 (SD 12, range 18–100). Univariate regression analysis found that older age (≥45 years) and having ever been married were statistically significantly related to lower HIV knowledge. Having a secondary education and living in an urban centre were statistically significantly related to higher knowledge. In a multivariate model containing age, education, sex, marital status, place of residence and having an HIV positive friend, having a secondary education or higher were the only significant predictors for HIV knowledge (Table 4).

**Table 4:** Univariate and multivariate linear regression analysis of HIV knowledge score

		**Univariate analysis**		**Multivariate analysis**	
**Variable**		**Slope (95%CI)**	**p**	**Slope (95%CI)**	**p**
Age	18-24	1.0		1.0	
	25-44	−1.02 (−4.74, 2.69)	0.510	0.91 (−3.08, 4.91)	0.582
	> = 45	−6.03(−11.77,-0.30)	**0.043**	−2.86 (−8.80,3.07)	0.270
Education	Primary or less	1.0		1.0	
	Secondary or higher	6.61 (3.15, 10.08)	**0.004**	0.94 (0.27,1.62)	**0.016**
House**	Mud walled	1.0		-	
	Concrete walled	0.43 (−4.92, 5.77)	0.846	-	
Sex	Male	1.0		1.0	
	Female	0.85 (−3.15, 4.84)	0.609	1.19 (−2.77, 5.14)	0.475
Marital status	Single	1.0		1.0	
	Ever married	−3.68 (−6.83, -0.54)	**0.030**	−1.87 (−5.05,1.31)	0.192
Residence	Rural	1.0		1.0	
	Urban	4.22 (0.50, 7.95)	**0.033**	2.00 (−1.75, 5.75)	0.229
Has an HIV positive friend	No	1.0		1.0	
	Yes	−2.62 (−6.90, 1.66)	0.176	−2.48 (−6.48,1.51)	0.171

### Comparison of TB and HIV knowledge scores

The mean TB knowledge percentage score was significantly lower than the HIV knowledge score with a difference in means of 24 (34 vs 58, p < 0.001). The most striking difference was that the most basic knowledge about TB such as transmission and curability was not known by most participants while the majority of respondents knew mode of transmission and treatment of HIV infection. In addition, the level of misconception was much higher for TB. For example, a number of respondents thought that TB can be “inherited” and it can be acquired through alcohol consumption, hard labour, or from air pollution. The major deficiency in HIV knowledge was the low awareness of mother-to-child-transmission of HIV which was the one reason for a lower HIV knowledge score than we expected. There were also major differences in the predictor variables for high knowledge in both infections (see Table 3 and 4). The minimum score in HIV knowledge was 18%, while seven participants had a zero knowledge score for TB. Three participants (0.8%) of participants had a HIV knowledge score of 18% and 50 (13.9%) of participants had a TB knowledge score of 18% or less.

## Discussion

This is the first time that TB knowledge has been assessed in this region. Our community study measured knowledge in ordinary villagers about this important infection in a typical region of sub-Saharan Africa. We also measured the HIV knowledge in the same survey and we were thus able to compare the community knowledge of both infections. We consider the results from this knowledge comparison to be important for policy makers who have to look at priorities for infectious disease programs in the context of limited district health budgets. The strength of our study was that we used a mixed design with quantitative and qualitative methods which enhanced the understanding on our topic and improved the validity of the findings. To the best of our knowledge, we could not find any published information from sub-Saharan Africa on a comparison between TB and HIV knowledge: therefore we consider our study as a contribution to the literature. Additionally, misconceptions about disease aetiology, treatment and prevention of TB and HIV are important to inform the delivery of effective TB and HIV treatment and prevention programs in the district. Service utilization and adherence to treatment and prevention protocols should ideally be based on these findings.

The TB knowledge in the population was unexpectedly low with a mean summary score of 33%. This result is classified as seriously deficient according to a district rating system suggested by Janowsky et al. and which is frequently used [14]. This low TB knowledge is corroborated by the additional finding that only 31% of study participants knew that TB is an airborne infection. Some of the “misconceptions” about TB transmission which we found were also reported in the other studies from eastern Uganda and elsewhere [7,11]. Sharing cups emerged as a very frequent response by participants trying to explain the transmission of TB in our study, but was not reported in the other studies [4,6,11,15]. Although this belief may have some truth in it (as saliva contamination could possibly transmit Mycobacterium tuberculosis from one person to another person when cups are shared without cleaning them, and two people sharing food and drinks are likely close enough for airborne transmission to occur), it distracts somewhat from the most important fact that the community must know that the major mode of TB transmission is airborne. This popular belief is not necessarily harmful as proper hygiene and not sharing cups and utensils lead generally to preventative health practice.

Surprising to us was the understanding by some that TB is an untreatable hereditary disease. This was also mentioned in each focus group discussion. It has also been described elsewhere in Uganda and in Tanzania [6,11]. This is a serious misconception and should be vigorously targeted in education programs, since conceptualising TB as a hereditary disease which cannot be treated has huge potential to negatively affect decisions to seek TB care. Another significant finding was that 20% of respondents did not know that TB can be cured which was also confirmed in the focus group discussions by participants. This has also serious implications for control: if people do not believe that their illness can be treated they will not seek care. This last finding is different from the other studies in Uganda, Tanzania, and Rwanda that found that people universally understood that TB was curable [6,9,11].

Older age and rural residence predicted higher TB knowledge. This is a surprising result, as we expected the opposite. The survey from Ethiopia on TB found that literacy predicted better knowledge of TB causation, transmission, and prevention [8]. As literacy is usually higher in younger people [16], our study finding of better TB knowledge in older persons is contrary to the findings from the Ethiopian study. Our hypothesis for an explanation of this finding is that TB education in the study area was a major focus within a Primary Health Care program in the mid 1980’s and 1990’s which was supported by the German Government and focused on rural areas in Kabarole District. As such, individuals who were old enough during that period to understand TB information presented then were positively impacted and increased their knowledge. Later on, this program was abandoned and priorities were given to HIV prevention and care. Therefore younger people may have remained uninformed about TB issues and consequently had a lower knowledge about TB. These previous program funding developments would also explain why TB knowledge was better in rural areas compared to urban areas, as one would expect the opposite.

HIV knowledge was much higher than TB knowledge with highly statistically significant differences (58% vs 34% knowledge, p < 0.001). The HIV knowledge was comparable to a survey conducted in 2005 in the same study area using a similar methodology and similar knowledge questions. In the 2005 survey the HIV knowledge in the general population was 59% [17]. Predictors of higher HIV knowledge were younger age and urban residence, similar to other studies which found that younger persons living in urban areas generally have better health knowledge than older persons residing in rural areas of sub-Saharan Africa. Knowledge about mother to child transmission of HIV was very low in our sample, with only 14% of respondents mentioning it as a means of HIV transmission, much lower than 75% of respondents reported by the study in Ethiopia [8]. This in line with other data from Kabarole District, where knowledge of mother-to-child transmission of HIV was found to be seriously deficient and the services of PMTCT (Prevention of Mother-to-Child Transmission of HIV) grossly underutilized [18].

The lower TB knowledge compared to the HIV knowledge was not unexpected. We suspect that it is a reflection of recent shifts in program development where there has been a major focus on antiretroviral treatment of HIV infection and a corresponding allocation of resources to HIV/AIDS programs. This shift of resources has been confirmed in a previous study in Kabarole, where it was found that human and financial resources were allocated to HIV/AIDS programs to the disadvantage of other communicable disease programs [19]. Health care workers in the study area could only informally confirm the poor management and outcomes of TB control in the district, as no hard reliable data is available. However, while we expected a lower knowledge of TB compared to HIV, we were surprised about the magnitude of the knowledge difference. TB’s status as a neglected infection [20,21] seems to be reinforced if we looked on community knowledge as an indicator.

The interpretation of responses for both the questionnaire and the FGDs pose some ambiguity, given that they can be evaluated either on a strictly scientific basis level or on a more inclusive approach, whereby peoples’ perceptions and beliefs are considered. An even more inclusive approach would be to consider the cultural factors of a particular population and develop a cultural model of health communication based on sound scientific information, community perceptions and the local culture. According to our experience from sub-Saharan Africa, this approach has rarely been selected. To achieve this, an interesting follow-up project could be undertaken here with participation of medical, public health and anthropological experts and a broad community involvement**.**

### Limitations

1. We used a non-standardized questionnaire; therefore, interpretation of the level of knowledge and its comparability with other studies is limited. However, the comparison of the TB and HIV knowledge score within our study can be considered valid as we used similar types of questions and the same methodology. The questionnaire results were reliable, as shown in the test-re-test agreement and valid when compared to the results from the qualitative study component which confirmed most of the quantitative results.

2. We cannot exclude interview bias and/or social desirability bias, as our study dealt with a sensitive topic. We minimized it by using highly trained interviewers with local knowledge of the culture and the language and supervised them closely during the data collection process.

3. The ambiguity of responses and how they are interpreted is acknowledged. In this study, we were leaning toward a more scientific evaluation of the responses, which may not be always the best approach to better health communication in communities.

4. Because participants were recruited during weekdays, there may be an under-representation of individuals employed in the formal sector since they would not have been home during the day. It was not possible within the time limits of the field work to include those individuals by making additional visits to the homes.

5. Predictors of the knowledge scores are based on cross-sectional data and therefore cannot be considered as causal.

## Conclusions

TB knowledge in Kabarole district is low and we suspect that it has declined in recent years. This paper documents that misconceptions around TB currently exist in this region. They need to be addressed through improved health education programs in order to improve TB treatment outcomes. In addition, more integrated TB/HIV care is needed, streamlining diagnosis and management of co-infected patients. Improved integration of TB and HIV services could also be a more cost-effective approach and a solution to dwindling funds. This is an urgent issue, as a recent study from nine sub-Saharan African countries showed that out of 663 HIV treatment sites, only 47% (range 2-77%) had written TB communication plans [22].

This study could also give important guidance to the Kabarole district health managers so that TB and possibly other infectious diseases do not continue to be neglected at the expense of HIV/AIDS programming. Clearly, TB control is not receiving the attention this deadly disease deserves, be it because of the related stigma, departmentalized and fragmented health care planning and budgeting, lack of political will at all levels and/or the public/health care worker fatigue for this longstanding problem in the region. This study points to the danger of complacency in promoting improved TB control and our recommendation is that accurate and comprehensive TB prevention and treatment information should be conveyed at every possible opportunity, wherever and whenever it presents. Public education, care provider support, adequate resource allocation, integrated program services and political lobbying are critical means to this end.

## Competing interests

The authors declare that they have no competing interests.

## Authors’ contributions

AW conceived of the study, designed the study, conducted the fieldwork, conducted the quantitative and qualitative analysis, and jointly drafted the manuscript (with WK). GJ contributed to the design of the quantitative portion of the study and assisted with quantitative analysis. SR contributed to the design of the qualitative portion of the study and assisted with qualitative analysis. AA helped with design of data collection methods, data cleaning, and preliminary quantitative analysis. TR helped with design of data collection methods and field logistics. WK conceived of the study, designed the study, assisted with analysis, and jointly drafted the manuscript (with AW). All authors read and approved the final manuscript.

## Pre-publication history

The pre-publication history for this paper can be accessed here:

http://www.biomedcentral.com/1472-698X/12/36/prepub
